# Investigation of *PTEN* promoter methylation in ameloblastoma

**DOI:** 10.4317/medoral.23498

**Published:** 2020-03-06

**Authors:** Puangwan Lapthanasupkul, Boworn Klongnoi, Apiwat Mutirangura, Nakarin Kitkumthorn

**Affiliations:** 1 DDS, MSc, PhD. Department of Oral and Maxillofacial Pathology, Faculty of Dentistry, Mahidol University, Bangkok, Thailand; 2 DDS, MD, DMD. Department of Oral and Maxillofacial Surgery, Faculty of Dentistry, Mahidol University, Bangkok, Thailand; 3 MD, PhD. Center of Excellence in Molecular Genetics of Cancer and Human Diseases, Department of Anatomy, Faculty of Medicine, Chulalongkorn University, Bangkok, Thailand; 4 DDS, PhD. Department of Oral Biology, Faculty of Dentistry, Mahidol University, Bangkok, Thailand

## Abstract

**Background:**

Phosphatase and tensin homolog (*PTEN*) acts as a tumor suppressor gene. Inactivation of *PTEN* has been reported in various types of cancers. *PTEN* promoter methylation possibly underlies *PTEN* inactivation, which results in tumorigenesis. The aim of this study was to investigate whether *PTEN* promoter methylation contributes to *PTEN* inactivation in ameloblastoma and its associated protein expression.

**Material and Methods:**

In total, 20 fresh-frozen ameloblastoma samples were evaluated for *PTEN* promoter methylation using methylation-specific polymerase chain reaction (MS-PCR). A subset of 10 paraffin-embedded ameloblastoma samples was examined for PTEN expression through immunohistochemistry. Four primary cultured ameloblastoma cells were investigated for *PTEN* promoter methylation and *PTEN* transcriptional expression via reverse transcription PCR.

**Results:**

*PTEN* promoter methylation was detected in 65% (13/20) of the ameloblastoma samples. Of 10 ameloblastoma samples, 4 exhibited reduced PTEN expression. Of 5 samples with methylated *PTEN*, 3 (60%) were associated with loss of PTEN expression. However, PTEN expression was detected in 4 (80%) of 5 samples with unmethylated *PTEN*. In addition, 3 (75%) of 4 primary ameloblastoma cell cultures exhibited an inverse correlation between *PTEN* promoter methylation and *PTEN* transcription level.

**Conclusions:**

*PTEN* promoter methylation is found in a number of ameloblastomas but not significantly correlated with loss of PTEN expression. Genetic or epigenetic mechanisms other than *PTEN* promoter methylation may contribute to *PTEN* inactivation in ameloblastoma tumor cells.

** Key words:**PTEN, promoter methylation, ameloblastoma.

## Introduction

Ameloblastoma is the most frequently encountered neoplasm arising from the epithelium of the tooth-forming apparatus. Although this tumor is benign, it exhibits locally invasive behavior and has a high risk of recurrence. Its macroscopic features range from completely solid to multicystic appearance. Its histopathological subtypes include follicular, plexiform, acanthomatous, granular cell, basal cell, and desmoplastic ameloblastomas. In rare cases, ameloblastoma may metastasize despite its benign histology; this type of ameloblastoma is termed as metastasizing ameloblastoma (1). Ameloblastic carcinoma, a malignant counterpart of ameloblastoma, is markedly rare, with only 100 cases reported to date; this tumor exhibits cytological features of malignancy and may or may not metastasize (2).

Phosphatase and tensin homolog (*PTEN*) is located on chromosome 10q23.3 and has been implicated in many familial and sporadic cancers (3,4). Deletions or somatic mutations in *PTEN* have been detected in many types of cancers, including prostate, breast, and brain cancer (3). Apart from genetic mutation, the epigenetic regulation of *PTEN* via differential methylation may contribute to its inactivation (5). Methylation of the *PTEN* promoter region has been reported in some types of cancers and has been suggested to be involved in tumorigenesis (6-8). In ameloblastic tumors, *PTEN* displayed high frequent allelic loss (62%) (9). In addition, *PTEN* has been reported to be completely absent in 33.3% of ameloblastoma cases (10). We hypothesized that *PTEN* promoter methylation results in decreased *PTEN* expression in this odontogenic tumor. The aim of this study was to examine *PTEN* expression and investigate whether *PTEN* promoter methylation contributes to *PTEN* inactivation in ameloblastoma.

## Material and Methods

- Sample recruitment

Fresh-frozen samples were obtained from 20 patients with ameloblastoma from the Department of Oral and Maxillofacial Surgery, Faculty of Dentistry, Mahidol University between January 2018 and January 2019. Some parts of the specimens were fixed in 10% buffered formalin for hematoxylin and eosin staining. Histopathological diagnosis of solid/multicystic ameloblastoma was performed by two oral pathologists (PL and NK). Furthermore, a cohort study was performed wherein 4 fresh solid/multicystic ameloblastoma tissue samples were harvested to form a primary cell culture. Table 1 displays the detailed demographic data.

**Table 1:**
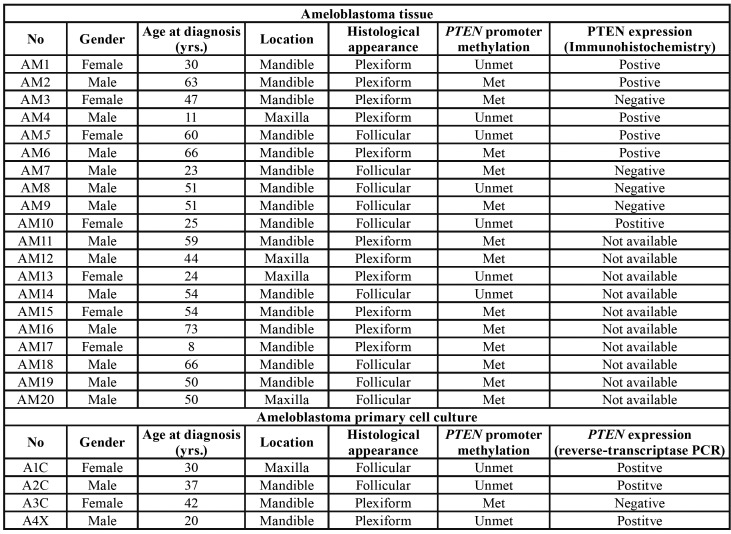
Detailed data of phosphatase and tensin homolog (<italic>*PTEN*</italic>) promoter methylation, *PTEN* expression in ameloblastoma and demographic data.

The cultures were grown in Dulbecco’s modified Eagle’s medium supplemented with 10% fetal bovine serum (Invitrogen, Carlsbad, CA, USA) and maintained for 3-4 passages prior to DNA extraction.

- DNA extraction, bisulfite modification, and methylation-specific polymerase chain reaction (MS-PCR)

Genomic DNA was extracted using 10% sodium dodecyl sulfate buffer and proteinase K at 50°C overnight, followed by phenol/chloroform extraction and ethanol precipitation. Subsequently, the obtained DNA was air dried, dissolved in distilled water, and quantified using a NanoDrop spectrophotometer (ND-1000 Spectrophotometer; NanoDrop Technologies, Wilmington, DE, USA). An optical density (at 260/280 ratio) of >1.8 is accepTable for DNA purity and PCR. Next, the DNA samples were converted by sodium bisulfite treatment using the EZ DNA Methylation-Gold kit (Zymo Research, Irvine, CA, USA), as per the manufacturer’s instructions.

The bisulfite-treated DNA samples were then subjected to MS-PCR using primers specific for either the methylated or unmethylated forms of *PTEN*: 1) *PTEN* methylated sequence, forward 5′-GTTTGGGGATTTTTTTTTCGC-3′ and reverse 5′-AACCCTTCCTACGCCGCG-3′ and 2) *PTEN* unmethylated sequence, forward 5′-TATTAGTTTGGGGATTTTTTTTTTGT-3′ and reverse 5′-CCCAACCCTTCCTACACCACA-3′ (11). Both forms of *PTEN* were amplified with HotStarTaq (Qiagen, Tokyo, Japan) in 40 cycles at an annealing temperature of 60°C. Aliquots of MS-PCR products (181 bp, both PCRs) were analyzed on 2% agarose gel or 8% nondenaturing acrylamide gel and then stained with SYBR green nucleic acid gel stain (Gelstar, Lonza, Allendale, NJ, USA).

For MS-PCR analysis, *PTEN* unmethylated primers amplified unmethylated product while *PTEN* methylated primers amplified only methylated product present in the ameloblastoma tissue. From our pilot study, DNA extracted from normal fibrous connective tissue was only amplified by unmethylated primers but not amplified by methylated primers. Thus, the methylated product, if present, came from ameloblastoma tumor cells.

In our pilot study, we examined DNA in different cell lines and observed that the HeLa cell line had partial *PTEN* promoter methylation and *PTEN* expression. Therefore, HeLa DNA and distilled water were included as the positive and negative controls, respectively, in all experiments.

- RNA extraction and reverse transcription PCR (RT-PCR)

*PTEN* expression in the ameloblastoma primary cell cultures was examined using RT-PCR. Total RNA was extracted using the TRIzol reagent (Invitrogen, Singapore), as per the manufacturer’s instructions. Single-stranded complementary DNA (cDNA) was synthesized using the RevertAid First Strand cDNA Synthesis kit (Thermo Fisher Scientific, Waltham, MA, USA), as per the manufacturer’s instructions.

*PTEN* was amplified using HotStarTaq (Qiagen, Tokyo, Japan) in 40 cycles at an annealing temperature of 55°C using the forward primer 5′-GGACGAACTGGTGTAATGATATG-3′ and reverse primer 5′-TCTACTGTTTTTGTGAAGTACAGC-3′ (12). To investigate the relative expression of a candidate gene, glyceraldehyde-3-phosphate dehydrogenase was used as an endogenous DNA control, with the sequences of the forward and reverse primers being 5′-CTCAGACACCATGGGGAAGGTGA-3′ and 5′-ATGATCTTGAGGCTGTTGTCATA-3′, respectively. Both PCR mixtures contained PCR buffer (1×) (Qiagen, Tokyo, Japan), deoxynucleotide triphosphates (0.2 mM), the two primers (final concentration: 0.4 μM), HotStarTaq (1 U) (Qiagen, Tokyo, Japan), and template DNA (50 ng). The PCR products (*PTEN*: 671 bp and GADPH: 450 bp) were separated via gel electrophoresis on an 8% nondenaturing acrylamide gel and stained with SYBR green nucleic acid gel stain (Gelstar, Lonza, Allendale, NJ, USA).

- Immunohistochemical staining of *PTEN*

In this experiment, the paraffin tissues were available in only 10 cases. Formalin-fixed paraffin embedded blocks were cut into 3-μm thick sections. The histological sections of the relative samples were confirmed by a pathologist. Immunohistochemistry was performed with an antihuman monoclonal antibody against *PTEN* Clone 6H2.1 (dilution 1:100, DAKO, Glostrup, Denmark) in Tris-HCl buffer antibody diluent (Dako, Glostrup, Denmark) on the Ventana® Benchmarck XT (Ventana-Roche Diagnostics, Meylan, France) automated slide strainer in combination with the Ventana UltraView DAB IHC Detection Kit®. Before mounting, the sections were counterstained with Hematoxylin II® for 8 min, bluing reagent® for 4 min, hematoxylin II for 4 min, and bluing reagent for 4 min. To support the validity of staining and identify experimental artifacts, negative (omitting the primary antibody) and positive controls (normal breast tissue) were included in each run. Nuclear and cytoplasmic immunostaining of *PTEN* in ameloblastoma tumor cells were graded based on the presence or absence of protein staining.

- Statistical analysis

SPSS software for Windows version 22 (SPSS Inc., Chicago, IL) was used to analyze all data. The effects of age and sex of the patients as well as the histological appearance of ameloblastoma on the *PTEN* methylation status and *PTEN* expression were investigated using the Pearson’s correlation coefficient test, chi-square test, and Fisher’s exact test. *P* < 0.05 was considered statistically significant.

## Results

- MS-PCR and immunohistochemistry of ameloblastoma tissues

**Figure 1:**
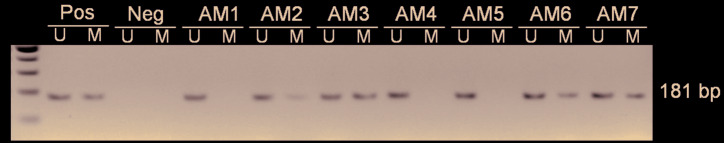
MS-PCR analysis of *PTEN* promoter in ameloblastoma samples. PCR products amplified using primers specific for unmethylated (U) and methylated (M) forms. AM2, AM3, AM6, and AM7 show promoter methylation. The ladder in the left lane is a 100-bp marker. Both methylated and unmethylated PCR products are 181 bp. Positive control, Pos: HeLa cell line; negative control, Neg: distilled water; unmethylated PCR products, U; methylated PCR products, M.

**Figure 2:**
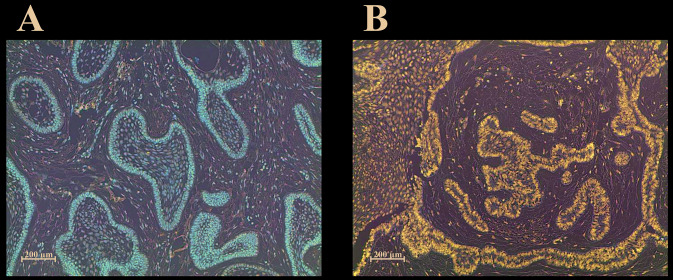
(A) Immunostaining of *PTEN* in ameloblastoma (streptavidin–biotin; 100×). *PTEN* immunoexpression is observed in cytoplasm and nucleus of ameloblastoma tumor cells (B) No expression of *PTEN* in ameloblastoma (streptavidin–biotin, 100×).

We examined *PTEN* promoter methylation and whether it affects *PTEN* expression in ameloblastomas. *PTEN* promoter methylation was observed in 65% (13/20) of ameloblastoma samples (Table 1). The exemplified gel electrophoresis is demonstrated in Fig. 1. Ten samples of these ameloblastoma cases were investigated for immunohistochemical staining of *PTEN*. We found loss of *PTEN* expression in 3 of 5 (60%) ameloblastoma samples with *PTEN* promoter methylation while *PTEN* expression was present in 4 of 5 (80%) ameloblastoma samples with no *PTEN* promoter methylation (Table 1). Representative samples showing positive and negative immunostaining of *PTEN* were shown in Fig. 2.

- Association among *PTEN* promoter methylation, *PTEN* expression, and clinicopathological parameters

Table 1 shows the association between *PTEN* promoter methylation and *PTEN* expression in the ameloblastoma samples. No significant correlation was found between *PTEN* promoter methylation and *PTEN* expression (*P* = 0.52). Furthermore, no correlation between *PTEN* promoter methylation and age (*P* = 0.49), gender (*P* = 0.40), location (*P* = 0.62) and the histological appearance of ameloblastoma (*P* = 0.41) was demonstrated. Similarly, no correlation was observed between *PTEN* expression and age (*P* = 0.25), gender (*P* = 1.00), location (*P* = 0.51), and the histological appearance of ameloblastoma (*P* = 1.00).

- MS-PCR and RT-PCR of primary ameloblastoma cell cultures

**Figure 3:**
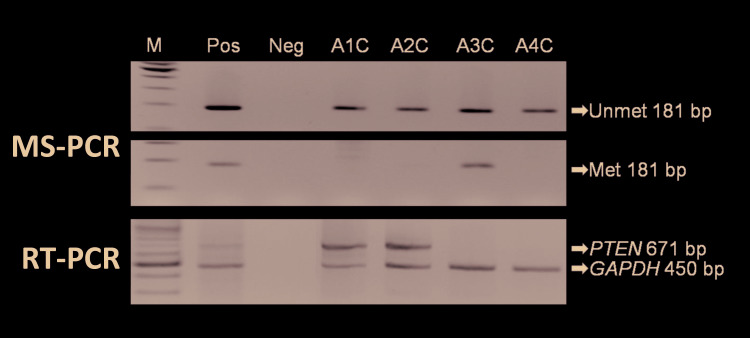
MS-PCR and RT-PCR analyses of promoter methylation and mRNA expression of <italic>*PTEN*</italic> in primary ameloblastoma cell cultures. The upper and middle panels show MS-PCR results, whereas the bottom panel shows RT-PCR results. Arrows indicate the locations of the expected amplicons. <italic>GAPDH</italic> was used in RT-PCR as an internal control. Standard 100 bp marker, M; positive control, Pos: HeLa cell line; negative control, Neg: distilled water; methylation-specific PCR, MS-PCR; reverse transcription polymerase chain reaction, RT-PCR; phosphatase and tensin homolog, <italic>*PTEN*</italic>; glyceraldehyde-3-phosphate dehydrogenase, <italic>GAPDH</italic>; unmethylated PCR products, Unmet; methylated PCR products, Met.

*PTEN* promoter methylation and *PTEN* expression were examined in four primary ameloblastoma cell cultures using RT-PCR (Fig. 3). Only 1 of 4 samples exhibited *PTEN* promoter methylation and showed no *PTEN* transcription. *PTEN* promoter methylation was inversely correlated with *PTEN* transcription level in the remaining ameloblastoma samples (75%) (Table 1).

## Discussion

Ameloblastoma is considered the most common benign neoplasm of the jaw (1). To prevent local recurrence, patients with ameloblastoma are mostly treated with radical surgery. Understanding the molecular mechanisms that underlie the formation of this tumor may help in developing an alternative and novel treatment for its cure with minimal tissue or bone removal.

*PTEN*, a putative tumor suppressor gene, is commonly mutated in many types of human neoplasms (3). The protein product of *PTEN*, a lipid phosphatase, negatively regulates the Akt signaling pathway, thereby stimulating cell cycle arrest and apoptosis (13). Kumamoto and Ooya first reported that the *PTEN* level is significantly lower in ameloblastic tumors than in tooth germs (14). The absence of *PTEN* in 33.3% of ameloblastoma samples was subsequently reported (10). These results suggest that the inactivation of *PTEN* may be involved in the molecular pathogenesis of ameloblastoma. In the present study, we investigated the possible role of *PTEN* promoter methylation and the associated loss of *PTEN* expression in a subset of ameloblastoma samples.

To the best of our knowledge, there have been no studies on *PTEN* promoter in ameloblastoma. Careful analysis of the *PTEN* promoter has been recommended because it shares a strong homology with the *PTEN* pseudogene (5). The genomic sequence of the highly conserved and processed *PTEN* pseudogene (GenBank accession number: AF040103, *PTEN* pseudogene; AF029308, Homo sapiens chromosome 9 duplication of the T-cell receptor β locus and trypsinogen gene families) is 98% identical to that of *PTEN*, and this identical sequence is composed of an 841-bp region in the promoter region (15). In the present study, *PTEN* promoter methylation was performed using methylation-specific primers that do not amplify the highly homologous *PTEN* pseudogene because these primers lie outside the sequence homology of the *PTEN* pseudogene.

Promoter methylation is reportedly one of the epigenetic mechanisms underlying the aberrant expression of tumor suppressor genes and contributing to the development of various types of cancers. For example, the methylation of adenomatous polyposis coli promoter is reportedly associated with tumor in the colon and breasts (16). *PTEN* promoter methylation is also observed in various types of cancers, including gastric, breast, colorectal, and lung cancer (7,8,17-20).

In the present study, *PTEN* promoter methylation was found in 65% (13/20) of the ameloblastoma samples. However, immunohistochemical staining of *PTEN* expression was performed in only 10 samples. Of these samples, 3 (60%) of 5 samples with *PTEN* promoter methylation were associated with loss of *PTEN* expression, whereas 4 (80%) of 5 samples without *PTEN* promoter methylation showed *PTEN* expression. *PTEN* promoter methylation and decreased *PTEN* expression were not significantly correlated, indicating that other genetic or epigenetic mechanisms possibly regulate *PTEN* expression, for example, genetic alterations, transcriptional silencing, post-transcriptional regulation, and modification (21). Previously, *PTEN* exhibited high frequency of allelic losses (62%) in ameloblastic tumors (9). Moreover, Narayan *et al*. reported that 5 (25%) of 20 samples of solid/multicystic ameloblastoma exhibited gene alterations in exon 5 of *PTEN* while no *PTEN* mutation was observed in normal tooth germs. However, associated protein expression was not examined in those samples (22). Based on the two-hit model (23), it may be possible that *PTEN* promoter methylation and allelic loss play a role in *PTEN* inactivation since *PTEN* is a tumor suppressor gene. It is also possible that *PTEN* promoter methylation, contributing to a decrease in protein expression, depends on the specific tumor type. Previous studies on lung and ovarian cancers did not see a correlation between *PTEN* promoter methylation and loss of protein expression (18,24).

Notably, in the present study, 2 (40%) of 5 samples with *PTEN* promoter methylation showed *PTEN* expression. This may be attributed to the partial methylation of *PTEN* at the promoter region. It has been proposed that translational inactivation involves a series of events requiring a sufficient DNA methylation level. The silencing process is then maintained by the spread of methylation (25). This is also supported by the presence of unmethylated bands in several samples following MS-PCR. However, these unmethylated bands also represent normal fibrous tissue stroma in the ameloblastoma samples.

Regarding the *in vitro* experiment, only 1 (25%) of 4 primary ameloblastoma cell cultures showed promoter methylation and loss of *PTEN* transcription. This result is consistent with a previous study on breast cancer; none of the breast cancer cell lines exhibited *PTEN* promoter methylation (17). Lastly, the limitation of the present study is the small sample size that may not represent ameloblastoma cases and cell lines in general; thus, further studies with a larger sample size are required to confirm our findings.

In conclusion, *PTEN* promoter methylation was detected in a subset (58.3%) of ameloblastoma samples; however, it did not significantly contribute to decreased *PTEN* expression. Other genetic mechanisms possibly underlie the loss of *PTEN* expression in ameloblastomas.
